# 3-Phenyl­diazen­yl-1,2-dimethyl-1*H*-indole

**DOI:** 10.1107/S1600536810031648

**Published:** 2010-08-18

**Authors:** Tuncer Hökelek, Nusret Tuna Biçer, Zeynel Seferoğlu, Ertan Şahin

**Affiliations:** a Department of Physics, Hacettepe University, 06800 Beytepe, Ankara, Turkey; b Department of Chemistry, Gazi University, 06500 Beşevler, Ankara, Turkey; c Department of Chemistry, Atatürk University, 22240 Erzurum, Turkey

## Abstract

In the title mol­ecule, C_16_H_15_N_3_, the indole ring system is planar within 0.021 (3) Å and the phenyl ring is inclined to this plane by 17.32 (14)°. π–π contacts involving the pyrrole rings of inversion-related indole units [centroid–centroid distance = 3.5187 (17) Å] stabilize the crystal structure.

## Related literature

For the use and applications of azo compounds, see: Bach *et al.* (1996 ▶); Bahatti & Seshadri (2004 ▶); Biswas & Umapathy (2000 ▶); Catino & Farris (1985 ▶); Clark & Hester (1993 ▶); Fadda *et al.* (1994 ▶); Hunger (2003 ▶); Taniike *et al.* (1996 ▶); Zollinger (2003 ▶); Willner & Rubin (1996 ▶). For related structures, see: Hökelek *et al.* (2007*a* ▶,*b* ▶); Seferoğlu *et al.* (2006 ▶, 2007 ▶, 2008 ▶). For standard bond lengths, see: Allen *et al.* (1987 ▶).
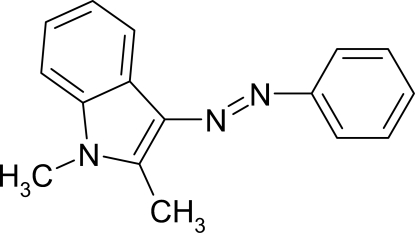


## Experimental

### 

#### Crystal data

C_16_H_15_N_3_
*M* *_r_* = 249.31
Monoclinic, 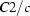

*a* = 16.3442 (3) Å
*b* = 10.2713 (2) Å
*c* = 16.5312 (3) Å
β = 104.264 (3)°
*V* = 2689.64 (9) Å^3^
*Z* = 8
Mo *K*α radiation
μ = 0.08 mm^−1^
*T* = 294 K
0.35 × 0.28 × 0.18 mm

#### Data collection

Rigaku R-AXIS RAPID-S diffractometer
27159 measured reflections
2762 independent reflections
1503 reflections with *I* > 2σ(*I*)
*R*_int_ = 0.103

#### Refinement

*R*[*F* ^2^ > 2σ(*F* ^2^)] = 0.066
*wR*(*F* ^2^) = 0.198
*S* = 1.04
2762 reflections
221 parameters
H atoms treated by a mixture of independent and constrained refinement
Δρ_max_ = 0.11 e Å^−3^
Δρ_min_ = −0.18 e Å^−3^

### 

Data collection: *CrystalClear* (Rigaku/MSC, 2005 ▶); cell refinement: *CrystalClear*; data reduction: *CrystalClear*; program(s) used to solve structure: *SHELXS97* (Sheldrick, 2008 ▶); program(s) used to refine structure: *SHELXL97* (Sheldrick, 2008 ▶); molecular graphics: *ORTEP-3 for Windows* (Farrugia, 1997 ▶); software used to prepare material for publication: *WinGX* publication routines (Farrugia, 1999 ▶) and *PLATON* (Spek, 2009 ▶).

## Supplementary Material

Crystal structure: contains datablocks I, global. DOI: 10.1107/S1600536810031648/su2202sup1.cif

Structure factors: contains datablocks I. DOI: 10.1107/S1600536810031648/su2202Isup2.hkl

Additional supplementary materials:  crystallographic information; 3D view; checkCIF report

## Figures and Tables

**Fig. 1.**
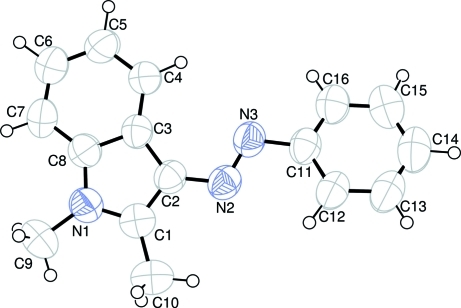
The molecular structure of the title molecule with the atom-numbering scheme. Displacement ellipsoids are drawn at the 50% probability level.

## supplementary crystallographic information

### Comment

Azo compounds are very important in the field of dyes, pigments and advanced materials (Hunger, 2003). It has been known for many years that the azo compounds are the most widely used class of dyes, due to their versatile applications in various fields such as the dyeing of textile fibers, the coloring of different materials, colored plastics and polymers, biological-medical studies and advanced applications in organic syntheses (Catino & Farris, 1985; Zollinger, 2003; Bahatti & Seshadri, 2004; Taniike *et al.*, 1996; Fadda *et al.*, 1994). They are also used in the fields of nonlinear optics and optical data storage (Taniike *et al.*, 1996; Bach *et al.*, 1996; Clark & Hester, 1993). Their optical properties depend on not only the spectroscopic properties of the molecules but also their crystallographic arrangements (Biswas & Umapathy, 2000; Willner & Rubin, 1996). Previously, the syntheses, crystal structures, spectroscopic and tautomeric properties of novel azo indole dyes have been reported in solution and solid state (Hökelek *et al.*, 2007*a*,*b*; Seferoğlu *et al.*, 2008; Seferoğlu *et al.*, 2007; Seferoğlu *et al.*, 2006). We report herein on the synthesis and crystal structure of the title compound.

The molecular structure of the title molecule is shown in Fig. 1. The bond lengths (Allen *et al.*, 1987) and angles are in normal ranges. The indole ring system is planar to within 0.022 (3) Å, with a dihedral angle of 1.22 (15)° between rings A (N1/C1-C3/C8) and B (C3-C8). The orientation of the phenyl ring C (C11-C16) with respect to the indole ring system may be described by the dihedral angle of 17.32 (14)°. Atoms C9, C10, N2 and N3 are displaced by 0.006 (3), -0.107 (4), 0.003 (2) and 0.050 (2) Å, respectively, from the plane of the indole ring system, hence are almost coplanar.

In the crystal there are π···π contacts involving A rings, of the indole group, related by an inversion center, which stabilize the crystal packing: Cg1—Cg1^i^ distance is 3.519 (2) Å [symmetry code: (i) -x, -y, -z, where Cg1 is the centroid of the pyrrole ring A (N1/C1-C3/C8)].

### Experimental

For the preparation of the title compound, aniline (190 mg, 2 mmol) was dissolved in HCl (1.5 ml) and water (4.0 ml). The solution was cooled in an ice-salt bath and a cold solution of NaNO_2_ (150 mg, 2 mmol) in water (3.0 ml) was added dropwise with stirring. The resulting diazonium salt was cooled in an ice-salt bath and then added dropwise with stirring to 1,2-dimethylindole (300 mg, 2 mmol) in an acetic acid/propionic acid mixture (2:1, 8.0 ml). The solution was stirred at 273-278 K for 1 h and the pH of the reaction mixture was maintained at 4-6 by the simultaneous addition of a sodium hydroxide solution (40-50 ml). The mixture was stirred for a further 1 h. The resulting solid was filtered, washed with cold water and crystallized from ethanol (yield; 440 mg, 92%, m.p. 398 K).

### Refinement

The C9 methyl H-atoms were included in calculated positions and treated as riding atoms: C—H = 0.96 Å, with *U*_iso_(H) = *1.5U*_eq_(C). The remaining H-atoms were located in a difference Fourier map and were refined freely.

### Crystal data

**Table:**

C_16_H_15_N_3_	*F*(000) = 1056
*M**_r_* = 249.31	*D*_x_ = 1.231 Mg m^−^^3^
Monoclinic, *C*2/*c*	Mo *K*α radiation, λ = 0.71073 Å
Hall symbol: -C 2yc	Cell parameters from 3396 reflections
*a* = 16.3442 (3) Å	θ = 2.4–26.4°
*b* = 10.2713 (2) Å	µ = 0.08 mm^−^^1^
*c* = 16.5312 (3) Å	*T* = 294 K
β = 104.264 (3)°	Block, orange
*V* = 2689.64 (9) Å^3^	0.35 × 0.28 × 0.18 mm
*Z* = 8	

### Data collection

**Table:**

Rigaku R-AXIS RAPID-S diffractometer	1503 reflections with *I* > 2σ(*I*)
Radiation source: fine-focus sealed tube	*R*_int_ = 0.103
graphite	θ_max_ = 26.4°, θ_min_ = 2.4°
ω scans	*h* = −20→20
27159 measured reflections	*k* = −12→12
2762 independent reflections	*l* = −20→18

### Refinement

**Table:**

Refinement on *F*^2^	Primary atom site location: structure-invariant direct methods
Least-squares matrix: full	Secondary atom site location: difference Fourier map
*R*[*F*^2^ > 2σ(*F*^2^)] = 0.066	Hydrogen site location: inferred from neighbouring sites
*wR*(*F*^2^) = 0.198	H atoms treated by a mixture of independent and constrained refinement
*S* = 1.04	*w* = 1/[σ^2^(*F*_o_^2^) + (0.0864*P*)^2^ + 0.4512*P*] where *P* = (*F*_o_^2^ + 2*F*_c_^2^)/3
2762 reflections	(Δ/σ)_max_ < 0.001
221 parameters	Δρ_max_ = 0.11 e Å^−^^3^
0 restraints	Δρ_min_ = −0.18 e Å^−^^3^

### Special details

**Table:**

Geometry. All esds (except the esd in the dihedral angle between two l.s. planes) are estimated using the full covariance matrix. The cell esds are taken into account individually in the estimation of esds in distances, angles and torsion angles; correlations between esds in cell parameters are only used when they are defined by crystal symmetry. An approximate (isotropic) treatment of cell esds is used for estimating esds involving l.s. planes.
Refinement. Refinement of F^2^ against ALL reflections. The weighted R-factor wR and goodness of fit S are based on F^2^, conventional R-factors R are based on F, with F set to zero for negative F^2^. The threshold expression of F^2^ > 2sigma(F^2^) is used only for calculating R-factors(gt) etc. and is not relevant to the choice of reflections for refinement. R-factors based on F^2^ are statistically about twice as large as those based on F, and R- factors based on ALL data will be even larger.

### Fractional atomic coordinates and isotropic or equivalent isotropic displacement parameters (Å^2^)

**Table:**

	*x*	*y*	*z*	*U*_iso_*/*U*_eq_	
N1	0.08081 (14)	0.0254 (2)	0.10187 (13)	0.0815 (7)	
N2	0.14870 (13)	0.1760 (2)	−0.06346 (13)	0.0776 (6)	
N3	0.12083 (14)	0.2792 (2)	−0.10430 (14)	0.0809 (6)	
C1	0.13054 (17)	0.0347 (3)	0.04696 (16)	0.0788 (7)	
C2	0.10816 (16)	0.1455 (3)	−0.00174 (16)	0.0735 (7)	
C3	0.04189 (16)	0.2101 (2)	0.02639 (15)	0.0740 (7)	
C4	−0.00515 (18)	0.3236 (3)	0.00533 (18)	0.0830 (8)	
H4	0.0013 (16)	0.382 (3)	−0.0413 (16)	0.091 (8)*	
C5	−0.06401 (19)	0.3556 (3)	0.0491 (2)	0.0941 (9)	
H5	−0.0962 (17)	0.443 (3)	0.0354 (16)	0.098 (8)*	
C6	−0.0776 (2)	0.2777 (3)	0.1129 (2)	0.1000 (10)	
H6	−0.1200 (19)	0.301 (3)	0.1470 (17)	0.105 (9)*	
C7	−0.03322 (19)	0.1639 (3)	0.13422 (18)	0.0891 (9)	
H7	−0.0458 (17)	0.109 (3)	0.1772 (16)	0.096 (9)*	
C8	0.02687 (17)	0.1317 (3)	0.09164 (15)	0.0765 (7)	
C9	0.0837 (2)	−0.0775 (3)	0.16314 (18)	0.1000 (10)	
H9A	0.0273	−0.1057	0.1615	0.150*	
H9B	0.1099	−0.0451	0.2178	0.150*	
H9C	0.1157	−0.1496	0.1504	0.150*	
C10	0.1983 (2)	−0.0607 (4)	0.0456 (3)	0.1006 (10)	
H101	0.177 (2)	−0.153 (4)	0.032 (2)	0.139 (13)*	
H102	0.239 (2)	−0.063 (3)	0.097 (2)	0.118 (12)*	
H103	0.232 (2)	−0.032 (3)	0.004 (2)	0.122 (12)*	
C11	0.16377 (17)	0.3083 (3)	−0.16766 (16)	0.0773 (7)	
C12	0.24027 (19)	0.2553 (3)	−0.17310 (19)	0.0845 (8)	
H12	0.2668 (19)	0.184 (3)	−0.1278 (18)	0.112 (10)*	
C13	0.2756 (2)	0.2934 (4)	−0.2364 (2)	0.1010 (10)	
H13	0.326 (2)	0.255 (3)	−0.2414 (18)	0.099 (9)*	
C14	0.2366 (3)	0.3836 (4)	−0.2952 (2)	0.1079 (11)	
H14	0.265 (3)	0.408 (4)	−0.341 (2)	0.158 (14)*	
C15	0.1604 (3)	0.4367 (4)	−0.2903 (2)	0.1051 (10)	
H15	0.1309 (17)	0.507 (3)	−0.3271 (18)	0.096 (9)*	
C16	0.1245 (2)	0.4012 (3)	−0.2262 (2)	0.0936 (9)	
H16	0.0750 (19)	0.438 (3)	−0.2170 (18)	0.102 (10)*	

### Atomic displacement parameters (Å^2^)

**Table:**

	*U*^11^	*U*^22^	*U*^33^	*U*^12^	*U*^13^	*U*^23^
N1	0.0829 (15)	0.0828 (15)	0.0780 (14)	0.0006 (12)	0.0181 (12)	0.0063 (11)
N2	0.0683 (13)	0.0804 (15)	0.0873 (14)	−0.0050 (11)	0.0252 (11)	−0.0059 (12)
N3	0.0752 (14)	0.0862 (15)	0.0865 (15)	−0.0040 (12)	0.0302 (12)	0.0007 (12)
C1	0.0758 (17)	0.0795 (17)	0.0772 (17)	−0.0019 (14)	0.0116 (14)	−0.0068 (14)
C2	0.0680 (15)	0.0758 (16)	0.0790 (16)	−0.0035 (13)	0.0222 (13)	−0.0027 (13)
C3	0.0739 (16)	0.0765 (17)	0.0744 (16)	−0.0045 (13)	0.0233 (13)	0.0015 (13)
C4	0.0842 (18)	0.0767 (18)	0.094 (2)	0.0042 (15)	0.0336 (15)	0.0079 (15)
C5	0.090 (2)	0.086 (2)	0.117 (2)	0.0084 (16)	0.0458 (18)	0.0033 (18)
C6	0.101 (2)	0.103 (2)	0.110 (2)	0.0037 (19)	0.051 (2)	0.0061 (19)
C7	0.091 (2)	0.105 (2)	0.0790 (18)	−0.0056 (18)	0.0354 (15)	0.0061 (16)
C8	0.0760 (16)	0.0780 (17)	0.0737 (16)	−0.0014 (14)	0.0150 (13)	0.0014 (13)
C9	0.115 (2)	0.092 (2)	0.0880 (19)	−0.0002 (17)	0.0153 (17)	0.0204 (16)
C10	0.093 (2)	0.091 (2)	0.112 (3)	0.0140 (19)	0.016 (2)	−0.004 (2)
C11	0.0759 (17)	0.0817 (17)	0.0782 (17)	−0.0150 (14)	0.0264 (14)	−0.0105 (14)
C12	0.0813 (19)	0.090 (2)	0.089 (2)	−0.0125 (16)	0.0355 (16)	−0.0158 (16)
C13	0.097 (2)	0.110 (2)	0.110 (3)	−0.016 (2)	0.053 (2)	−0.023 (2)
C14	0.117 (3)	0.118 (3)	0.100 (3)	−0.034 (2)	0.048 (2)	−0.013 (2)
C15	0.114 (3)	0.109 (3)	0.092 (2)	−0.023 (2)	0.025 (2)	0.006 (2)
C16	0.088 (2)	0.101 (2)	0.095 (2)	−0.0115 (18)	0.0307 (18)	−0.0004 (18)

### Geometric parameters (Å, °)

**Table:**

N1—C1	1.363 (3)	C8—C7	1.382 (4)
N1—C8	1.388 (3)	C9—H9A	0.9600
N1—C9	1.457 (3)	C9—H9B	0.9600
N2—C2	1.382 (3)	C9—H9C	0.9600
N3—N2	1.279 (3)	C10—H101	1.02 (4)
N3—C11	1.429 (3)	C10—H102	0.93 (3)
C1—C10	1.482 (4)	C10—H103	1.03 (3)
C2—C1	1.390 (4)	C11—C16	1.397 (4)
C3—C2	1.442 (4)	C12—C11	1.387 (4)
C3—C4	1.393 (4)	C12—C13	1.371 (4)
C3—C8	1.415 (3)	C12—H12	1.06 (3)
C4—C5	1.378 (4)	C13—C14	1.380 (5)
C4—H4	1.00 (3)	C13—H13	0.93 (3)
C5—H5	1.04 (3)	C14—C15	1.381 (5)
C6—C5	1.384 (4)	C14—H14	1.01 (4)
C6—H6	1.02 (3)	C15—H15	0.99 (3)
C7—C6	1.375 (4)	C16—C15	1.381 (4)
C7—H7	0.96 (3)	C16—H16	0.94 (3)
			
C1—N1—C8	109.2 (2)	N1—C9—H9B	109.5
C1—N1—C9	126.3 (2)	N1—C9—H9C	109.5
C8—N1—C9	124.5 (2)	H9A—C9—H9B	109.5
N3—N2—C2	113.8 (2)	H9A—C9—H9C	109.5
N2—N3—C11	112.7 (2)	H9B—C9—H9C	109.5
N1—C1—C2	109.2 (2)	C1—C10—H102	112 (2)
N1—C1—C10	122.2 (3)	C1—C10—H101	114 (2)
C2—C1—C10	128.6 (3)	C1—C10—H103	110.4 (18)
N2—C2—C1	120.5 (2)	H102—C10—H101	108 (3)
N2—C2—C3	132.0 (2)	H102—C10—H103	104 (3)
C1—C2—C3	107.5 (2)	H101—C10—H103	109 (3)
C4—C3—C2	135.8 (2)	C12—C11—N3	125.3 (3)
C4—C3—C8	118.6 (2)	C12—C11—C16	119.4 (3)
C8—C3—C2	105.6 (2)	C16—C11—N3	115.2 (3)
C3—C4—H4	122.5 (15)	C11—C12—H12	116.3 (17)
C5—C4—C3	118.8 (3)	C13—C12—C11	119.5 (3)
C5—C4—H4	118.7 (15)	C13—C12—H12	124.2 (17)
C4—C5—C6	121.7 (3)	C12—C13—C14	121.5 (4)
C4—C5—H5	118.3 (15)	C12—C13—H13	119.7 (19)
C6—C5—H5	119.9 (15)	C14—C13—H13	118.8 (18)
C5—C6—H6	122.8 (16)	C13—C14—C15	119.4 (3)
C7—C6—C5	120.9 (3)	C13—C14—H14	118 (2)
C7—C6—H6	116.3 (16)	C15—C14—H14	122 (2)
C6—C7—C8	117.9 (3)	C14—C15—H15	124.3 (17)
C6—C7—H7	119.2 (17)	C16—C15—C14	120.0 (4)
C8—C7—H7	122.9 (17)	C16—C15—H15	115.4 (17)
N1—C8—C3	108.5 (2)	C11—C16—H16	115.3 (18)
C7—C8—N1	129.5 (3)	C15—C16—C11	120.2 (4)
C7—C8—C3	122.0 (3)	C15—C16—H16	124.5 (18)
N1—C9—H9A	109.5		
			
C8—N1—C1—C2	−1.7 (3)	C8—C3—C2—C1	−0.7 (3)
C8—N1—C1—C10	176.5 (3)	C2—C3—C4—C5	−178.6 (3)
C9—N1—C1—C2	179.3 (2)	C8—C3—C4—C5	0.4 (4)
C9—N1—C1—C10	−2.4 (4)	C2—C3—C8—N1	−0.3 (3)
C1—N1—C8—C3	1.2 (3)	C2—C3—C8—C7	179.8 (2)
C1—N1—C8—C7	−178.9 (3)	C4—C3—C8—N1	−179.6 (2)
C9—N1—C8—C3	−179.8 (2)	C4—C3—C8—C7	0.5 (4)
C9—N1—C8—C7	0.0 (4)	C3—C4—C5—C6	−0.2 (5)
N3—N2—C2—C1	178.9 (2)	C7—C6—C5—C4	−0.9 (5)
N3—N2—C2—C3	−1.8 (4)	C8—C7—C6—C5	1.8 (5)
C11—N3—N2—C2	−179.98 (19)	N1—C8—C7—C6	178.6 (3)
N2—N3—C11—C12	−15.8 (4)	C3—C8—C7—C6	−1.6 (4)
N2—N3—C11—C16	166.1 (2)	N3—C11—C16—C15	−179.9 (3)
N2—C2—C1—N1	−179.0 (2)	C12—C11—C16—C15	2.0 (4)
N2—C2—C1—C10	2.9 (4)	C11—C12—C13—C14	−0.1 (5)
C3—C2—C1—N1	1.5 (3)	C13—C12—C11—N3	−178.8 (2)
C3—C2—C1—C10	−176.6 (3)	C13—C12—C11—C16	−0.9 (4)
C4—C3—C2—N2	−1.0 (5)	C13—C14—C15—C16	1.1 (5)
C4—C3—C2—C1	178.4 (3)	C12—C13—C14—C15	0.0 (5)
C8—C3—C2—N2	179.9 (2)	C11—C16—C15—C14	−2.1 (5)
